# Crystal structures of two triazola-dioxola-benzena­cyclo­nona­phanes

**DOI:** 10.1107/S205698901501141X

**Published:** 2015-06-24

**Authors:** Vijayan Viswanathan, Naga Siva Rao, Raghavachary Raghunathan, Devadasan Velmurugan

**Affiliations:** a Centre of Advanced Study in Crystallography and Biophysics, University of Madras, Guindy Campus, Chennai 600 025, India; b Department of Organic Chemistry, University of Madras, Guindy Campus, Chennai 600 025, India

**Keywords:** crystal structure, triazole, dioxalabenzena­cyclo­nona­phane, pyrrolizine, pyrrolo­thia­zole, tetra­hydro­durodioxole, chiral, hydrogen bonding

## Abstract

In the title compounds, C_25_H_29_BrN_5_O_7_, (I) [systematic name: (*Z*)-1^5^-bromo-3^2^,3^2^-dimethyl-2^1^-nitro-2^2^,2^3^,2^5^,2^6^,2^7^,2^7a^,3^3a^,3^5^,3^6^,3^6a^-deca­hydro-2^1^ *H*,6^1^ *H*-4,9-dioxa-2(3,2)-pyrrolizina-6(4,1)-triazola-3(5,6)-furo[2,3-*d*][1,3]dioxola-1(1,2)-benzena­cyclo­nona­phane], and C_24_H_29_N_5_O_7_S, (II) [systematic name: (*Z*)-3^2^,3^2^-dimethyl-2^7^-nitro-2^5^,2^6^,2^7^,2^7a^,3^3a^,3^5^,3^6^,3^6a^-octa­hydro-2^1^ *H*,2^3^ *H*,6^1^ *H*-4,9-dioxa-2(5,6)-pyrrolo­[1,2-*c*]thia­zola-6(4,1)-triazola-3(5,6)-furo[2,3-*d*][1,3]dioxola-1(1,2)-benzena­cyclo­nona­phane], the triazole rings adopt almost planar conformations. In (I), the fused pyrrolidine rings adopt envelope conformations with the C atoms opposite the fused N—C bond as the flap in each ring, and their mean planes are inclined to one another by 52.8 (3)°. In (II), the pyrrolidine and thia­zole rings are both twisted on the fused N—C bond, and their mean planes are inclined to one another by 70.8 (2)°. In both (I) and (II), the furan ring adopts an envelope conformation with the adjacent C atom of the macrocycle as the flap. In the crystal of (I), mol­ecules are linked *via* C—H⋯N and C—H⋯O hydrogen bonds, forming sheets parallel to (10-1), while in (II), mol­ecules are linked *via* C—H⋯N and C—H⋯O hydrogen bonds, forming helical chains propagating along [010], which are linked *via* C—H⋯S hydrogen bonds, forming slabs parallel to (001).

## Abstract 2

Two dioxala-benzena­cyclo­nona­phanes differ principally by the presence of a pyrrolizine ring system in one and a pyrrolo­thia­zole ring system in the other. In the crystal of the former, mol­ecules are linked *via* C—H⋯N and C—H⋯O hydrogen bonds, forming sheets parallel to (10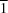) while in the latter, mol­ecules are linked *via* C—H⋯N, C—H⋯O and C—H⋯S hydrogen bonds, forming slabs parallel to (001).

## Chemical context   

Triazoles and their derivatives are of great importance in medicinal chemistry and can be used for the synthesis of many heterocyclic compounds with different biological activities such as anti­viral, anti­bacterial, anti­fungal (Mange *et al.*, 2013 ▸), anti­cancer (Singhal *et al.*, 2011 ▸), anti­tuberculosis, anti­convulsant, anti­depressant (Sahin *et al.*, 2012 ▸) and anti-inflammatory activities. They have been reported to be inhib­itors of glycogen synthase kinase-3, antagonists of GABA receptors, agonists of muscarine receptors and have been shown to possess anti-HIV-1, cytotoxic, anti­histaminic and anti­proliferative activities (Pokhodylo *et al.*, 2013 ▸). Triazoles are stable to acid and basic hydrolysis and reductive and oxidative conditions because of their high aromatic stabilization. In addition, this heterocycle has a high dipole moment and might participate in hydrogen-bond formation as well as in dipole–dipole and π-stacking inter­actions (Pertino *et al.*, 2013 ▸).

## Structural commentary   

**Figure 1:**
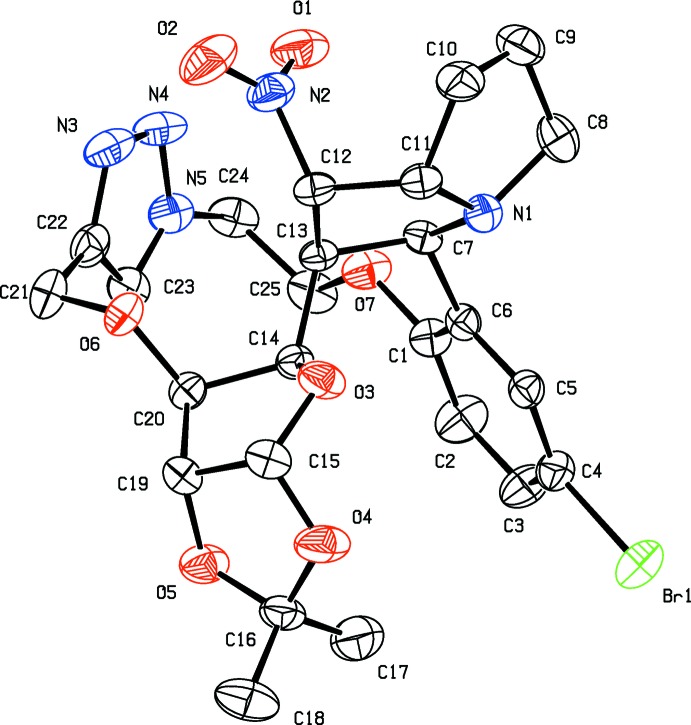
The mol­ecular structure of compound (I), showing the atom labelling. Displacement ellipsoids are drawn at the 30% probability level. H atoms are omitted for clarity.

The molecular structures of compounds (I) and (II) are illustrated in Figs. 1 ▸ and 2 ▸, respectively. The triazole rings (*A* = N3–N5/C22/C23) adopt almost planar conformations in both compounds. In compound (I), the pyrrolidine rings (*D* = N1/C11–C13/C7 and *E* = N1/C8–C11) and the furan ring (*B* = O3/C15/C19/C20/C14) have envelope conformations with the maximum deviations from the respective mean planes of 0.465 (5) Å for atom C13, 0.490 (7) Å for C9 and 0.500 (4) Å for C14. The dioxalane ring (*C* = O4/C15/C19/O5/C16) has a twisted conformation on bond O5—C15. The mean planes of rings *B* and *C* are inclined to one another by 70.0 (3)°, and the mean planes of rings *D* and *E* are inclined to one another by 52.8 (3)°. 
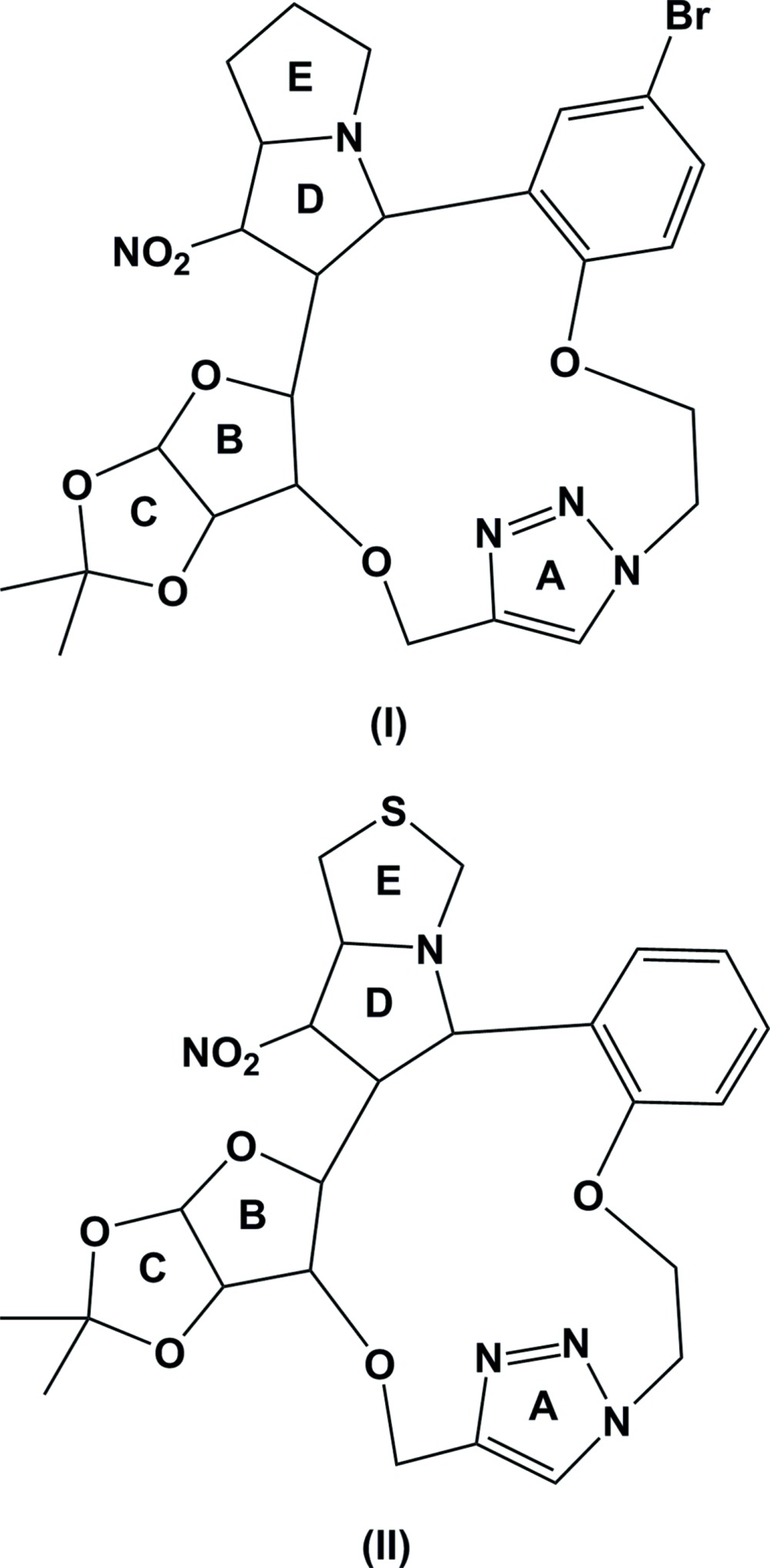


In compound (II), the pyrrolidine (*D*) and thia­zole rings (*E* = N1/C8/S9/C10/C11) have twist conformations on bond N1—C11. The furan and dioxolane rings (*B* and *C*) adopt envelope conformations with maximum deviations from the mean planes of 0.631 (3) Å for atom C14 and 0.319 (4) Å for C16. The mean planes of rings *B* and *C* are inclined to one another by 68.5 (2)° and the mean planes of rings *D* and *E* are inclined to one another by 70.8 (2)°. This latter dihedral angle is much larger than that in compound (I), *cf.* 52.8 (3)°.

In compound (I), the triazole ring (*A*) makes dihedral angles of 74.0 (3), 65.8 (3) and 65.8 (3)° with the mean planes of rings *B* and *D* and the benzene ring (C1–C6), respectively. The corresponding dihedral angles in compound (II) are 51.9 (2), 37.1 (2) and 60.9 (2)°, respectively. The most notable differences between the compounds involve dihedral angles *A*/*B* and *A*/*D*; 74.0 (3) and 65.8 (3), respectively, for (I[Chem scheme1]), and 51.9 (2) and 37.1 (2)°, respectively, for (II[Chem scheme1]).

## Supra­molecular features   

**Figure 3:**
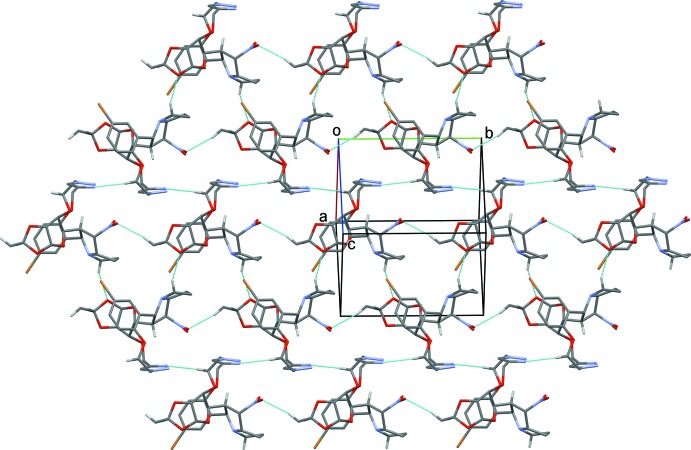
The crystal packing of compound (I), viewed approximately normal to plane (10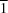). H atoms not involved in hydrogen bonding (dashed lines; Table 1 ▸) have been excluded for clarity.

**Figure 4:**
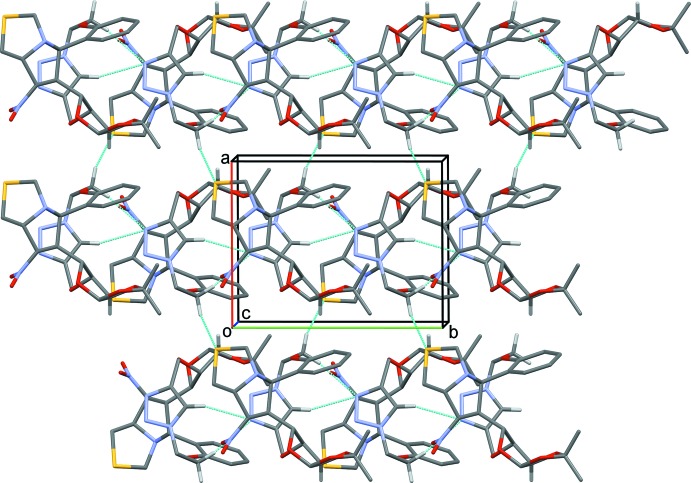
A view along the *c* axis of the crystal packing of compound (II), showing the hydrogen-bonded helical chains along [010], linked by C—H⋯S hydrogen bonds forming slabs parallel to the *ab* plane. H atoms not involved in hydrogen bonding (dashed lines; Table 2 ▸) have been excluded for clarity.

In the crystal of (I), mol­ecules are linked *via* C—H⋯N and C—H⋯O hydrogen bonds, forming sheets parallel to (10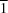); Table 1 ▸ and Fig. 3 ▸. In the crystal of (II), mol­ecules are linked *via* C—H⋯N and C—H⋯O hydrogen bonds, forming helical chains propagating along [010], which are linked *via* C—H⋯S hydrogen bonds, forming slabs parallel to (001); Table 2 ▸ and Fig. 4 ▸.

## Synthesis and crystallization   

**Compound (I)**: A solution of 5-bromo-2-(2-{4-[({(3a*S*,6*R*,6a*S*)-2,2-dimethyl-5-[(*Z*)-2-nitro­vin­yl]tetra­hydro­furo[2,3-*d*][1,3]di­oxol-6-yl}­oxy)meth­yl]-1*H*-1,2,3-triazol-1-yl}eth­oxy)benzalde­hyde (1 mmol) and proline (1.5 mmol) was refluxed in dry aceto­nitrile (50 ml) under a nitro­gen atmosphere for 9 h. After completion of the reaction, as indicated by TLC, the aceto­nitrile was evaporated under reduced pressure. The crude product was purified by column chromatography using hexa­ne/EtOAc (3:7) as eluent (yield 75%). After purification the compound was recrystallized in CHCl_3_ by slow evaporation yielding colourless block-like crystals.

**Compound (II)**: A solution of 5-bromo-2-(2-{4-[({(3a*S*,6*R*,6a*S*)-2,2-dimethyl-5-[(*Z*)-2-nitro­vin­yl]tetra­hydro­furo[2,3-*d*][1,3]dioxol-6-yl}­oxy)meth­yl]-1*H*-1,2,3-triazol-1-yl}eth­oxy)benzalde­hyde (1 mmol) and thia­zolidine-4-carb­oxy­lic acid (1.5 m mol) was refluxed in dry aceto­nitrile (50 ml) under a nitro­gen atmosphere for 9 h. After completion of reaction, as indicated by TLC,the aceto­nitrile was evaporated under reduced pressure. The crude product was purified by column chromatography using hexa­ne/EtOAc (4:6) as eluent (yield 75%). After purification the compound was recrystallized in CHCl_3_ by slow evaporation yielding colourless block-like crystals.

## Refinement   

Crystal data, data collection and structure refinement details are summarized in Table 3 ▸. The H atoms were placed in calculated positions and refined as riding: C—H = 0.93–0.98 Å with *U*_iso_(H) = 1.5*U*_eq_(C) for methyl H atoms and 1.2*U*_eq_(C) for other H atoms. Compound (I) was refined using the instructions TWIN/BASF (see Table 3 ▸).

## Supplementary Material

Crystal structure: contains datablock(s) global, I, II. DOI: 10.1107/S205698901501141X/su5103sup1.cif

Structure factors: contains datablock(s) I. DOI: 10.1107/S205698901501141X/su5103Isup2.hkl

Structure factors: contains datablock(s) II. DOI: 10.1107/S205698901501141X/su5103IIsup3.hkl

CCDC references: 1023614, 1023839

Additional supporting information:  crystallographic information; 3D view; checkCIF report

## Figures and Tables

**Figure 2:**
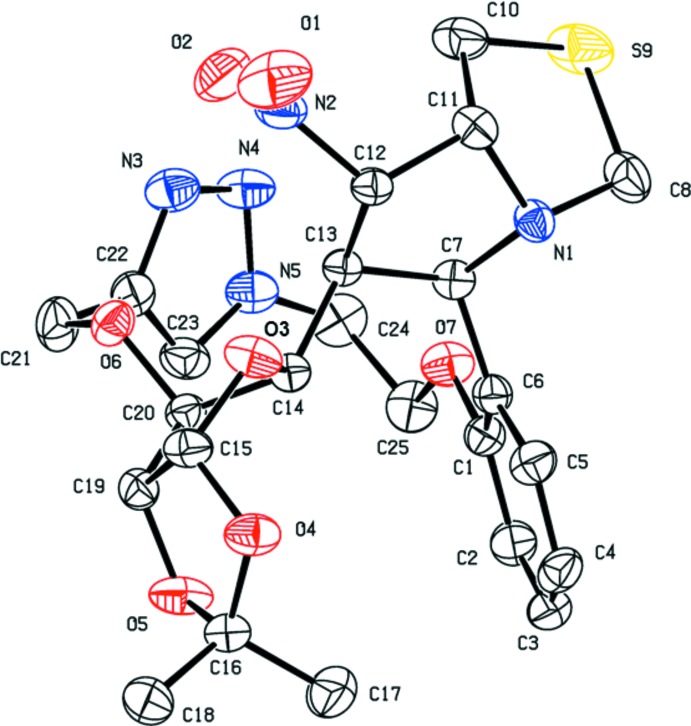
The mol­ecular structure of compound (II), showing the atom labelling. Displacement ellipsoids are drawn at the 30% probability level. H atoms are omitted for clarity.

**Table 1:** Hydrogen-bond geometry (Å, °) for (I)

*D*—H⋯*A*	*D*—H	H⋯*A*	*D*⋯*A*	*D*—H⋯*A*
C8—H8*B*⋯O4^i^	0.97	2.51	3.295 (7)	138
C18—H18*C*⋯O2^ii^	0.96	2.57	3.509 (9)	164
C25—H25*A*⋯N3^iii^	0.97	2.62	3.589 (7)	173

Symmetry codes: (i) 
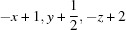
; (ii) 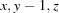; (iii) 
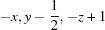
.

**Table 2:** Hydrogen-bond geometry (Å, °) for (II)

*D*—H⋯*A*	*D*—H	H⋯*A*	*D*⋯*A*	*D*—H⋯*A*
C23—H23⋯N3^i^	0.93	2.58	3.433 (6)	152
C25—H25*A*⋯N3^i^	0.97	2.60	3.553 (6)	168
C25—H25*B*⋯S9^ii^	0.97	2.80	3.591 (4)	140

Symmetry codes: (i) 
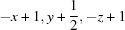
; (ii) 
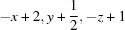
.

**Table 3:** Experimental details

	(I)	(II)
Crystal data		
Chemical formula	C_25_H_29_BrN_5_O_7_	C_24_H_29_N_5_O_7_S
*M*_r_	591.44	531.58
Crystal system, space group	Monoclinic, *P*2_1_	Monoclinic, *P*2_1_
Temperature (K)	293	293
*a*, *b*, *c* (Å)	9.913 (5), 11.414 (5), 12.144 (5)	8.756 (5), 10.811 (5), 13.569 (5)
β (°)	99.903 (5)	101.122 (5)
*V* (Å^3^)	1353.6 (11)	1260.3 (10)
*Z*	2	2
Radiation type	Mo *K*α	Mo *K*α
μ (mm^−1^)	1.57	0.18
Crystal size (mm)	0.20 × 0.15 × 0.10	0.20 × 0.15 × 0.10

Data collection		
Diffractometer	Bruker SMART APEXII area detector	Bruker SMART APEXII area detector
Absorption correction	Multi-scan (*SADABS*; Bruker, 2008 ▸)	Multi-scan (*SADABS*; Bruker, 2008 ▸)
*T*_min_, *T*_max_	0.744, 0.859	0.964, 0.982
No. of measured, independent and observed [*I* > 2σ(*I*)] reflections	12444, 6278, 3587	11813, 4712, 2862
*R*_int_	0.040	0.041
(sin θ/λ)_max_ (Å^−1^)	0.669	0.667

Refinement		
*R*[*F* ^2^ > 2σ(*F* ^2^)], *wR*(*F* ^2^), *S*	0.044, 0.105, 0.95	0.046, 0.103, 1.00
No. of reflections	6278	4712
No. of parameters	346	336
No. of restraints	1	1
H-atom treatment	H-atom parameters constrained	H-atom parameters constrained
Δρ_max_, Δρ_min_ (e Å^−3^)	0.58, −0.46	0.17, −0.24
Absolute structure	Refined as an inversion twin.	Flack *x* determined using 794 quotients [(*I* ^+^)−(*I* ^−^)]/[(*I* ^+^)+(*I* ^−^)] (Parsons *et al.*, 2013 ▸)
Absolute structure parameter	−0.007 (11)	−0.10 (9)

Computer programs: *APEX2* and *SAINT* (Bruker, 2008 ▸), *SHELXS97* (Sheldrick, 2008 ▸), *SHELXL2014* (Sheldrick, 2015 ▸), *PLATON* (Spek, 2009 ▸) and *Mercury* (Macrae *et al.*, 2008 ▸).

## supplementary crystallographic information

### (I) (Z)-15-Bromo-32,32-dimethyl-21-nitro-22,23,25,26,27,27a,33a,35,36,36a-decahydro-21H,61H-4,9-dioxa-2(3,2)-pyrrolizina-6(4,1)-triazola-3(5,6)-furo[2,3-d][1,3]dioxola-1(1,2)-benzenacyclononaphane Crystal data

**Table:**

C_25_H_29_BrN_5_O_7_	*F*(000) = 610
*M**_r_* = 591.44	*D*_x_ = 1.451 Mg m^−^^3^
Monoclinic, *P*2_1_	Mo *K*α radiation, λ = 0.71073 Å
*a* = 9.913 (5) Å	Cell parameters from 6278 reflections
*b* = 11.414 (5) Å	θ = 1.7–28.4°
*c* = 12.144 (5) Å	µ = 1.57 mm^−^^1^
β = 99.903 (5)°	*T* = 293 K
*V* = 1353.6 (11) Å^3^	Block, colourless
*Z* = 2	0.20 × 0.15 × 0.10 mm

### (I) (Z)-15-Bromo-32,32-dimethyl-21-nitro-22,23,25,26,27,27a,33a,35,36,36a-decahydro-21H,61H-4,9-dioxa-2(3,2)-pyrrolizina-6(4,1)-triazola-3(5,6)-furo[2,3-d][1,3]dioxola-1(1,2)-benzenacyclononaphane Data collection

**Table:**

Bruker SMART APEXII area-detector diffractometer	6278 independent reflections
Radiation source: fine-focus sealed tube	3587 reflections with *I* > 2σ(*I*)
Graphite monochromator	*R*_int_ = 0.040
ω and φ scans	θ_max_ = 28.4°, θ_min_ = 1.7°
Absorption correction: multi-scan (*SADABS*; Bruker, 2008)	*h* = −12→13
*T*_min_ = 0.744, *T*_max_ = 0.859	*k* = −14→15
12444 measured reflections	*l* = −16→16

### (I) (Z)-15-Bromo-32,32-dimethyl-21-nitro-22,23,25,26,27,27a,33a,35,36,36a-decahydro-21H,61H-4,9-dioxa-2(3,2)-pyrrolizina-6(4,1)-triazola-3(5,6)-furo[2,3-d][1,3]dioxola-1(1,2)-benzenacyclononaphane Refinement

**Table:**

Refinement on *F*^2^	Hydrogen site location: inferred from neighbouring sites
Least-squares matrix: full	H-atom parameters constrained
*R*[*F*^2^ > 2σ(*F*^2^)] = 0.044	*w* = 1/[σ^2^(*F*_o_^2^) + (0.0281*P*)^2^] where *P* = (*F*_o_^2^ + 2*F*_c_^2^)/3
*wR*(*F*^2^) = 0.105	(Δ/σ)_max_ < 0.001
*S* = 0.95	Δρ_max_ = 0.57 e Å^−^^3^
6278 reflections	Δρ_min_ = −0.46 e Å^−^^3^
346 parameters	Absolute structure: Refined as an inversion twin.
1 restraint	Absolute structure parameter: −0.007 (11)

### (I) (Z)-15-Bromo-32,32-dimethyl-21-nitro-22,23,25,26,27,27a,33a,35,36,36a-decahydro-21H,61H-4,9-dioxa-2(3,2)-pyrrolizina-6(4,1)-triazola-3(5,6)-furo[2,3-d][1,3]dioxola-1(1,2)-benzenacyclononaphane Special details

**Table:**

Geometry. All esds (except the esd in the dihedral angle between two l.s. planes) are estimated using the full covariance matrix. The cell esds are taken into account individually in the estimation of esds in distances, angles and torsion angles; correlations between esds in cell parameters are only used when they are defined by crystal symmetry. An approximate (isotropic) treatment of cell esds is used for estimating esds involving l.s. planes.

### (I) (Z)-15-Bromo-32,32-dimethyl-21-nitro-22,23,25,26,27,27a,33a,35,36,36a-decahydro-21H,61H-4,9-dioxa-2(3,2)-pyrrolizina-6(4,1)-triazola-3(5,6)-furo[2,3-d][1,3]dioxola-1(1,2)-benzenacyclononaphane Fractional atomic coordinates and isotropic or equivalent isotropic displacement parameters (Å^2^)

**Table:**

	*x*	*y*	*z*	*U*_iso_*/*U*_eq_	
Br1	0.67368 (6)	−0.19346 (6)	0.79878 (5)	0.0761 (2)	
O1	0.1833 (5)	0.4138 (4)	0.6920 (4)	0.0819 (13)	
O2	0.0613 (5)	0.4254 (4)	0.8212 (5)	0.0979 (16)	
O3	0.2313 (4)	0.0525 (3)	0.9095 (3)	0.0615 (10)	
O4	0.2390 (5)	−0.1342 (4)	0.9764 (4)	0.0874 (14)	
O5	0.0701 (3)	−0.1835 (4)	0.8366 (3)	0.0654 (9)	
O6	−0.0509 (3)	0.1090 (3)	0.7772 (3)	0.0521 (9)	
O7	0.2747 (4)	0.1097 (3)	0.4964 (3)	0.0565 (9)	
N1	0.4472 (4)	0.2353 (4)	0.8095 (3)	0.0452 (10)	
N2	0.1474 (5)	0.3774 (4)	0.7764 (5)	0.0640 (14)	
N3	−0.0839 (5)	0.2712 (4)	0.5621 (4)	0.0660 (13)	
N4	−0.0173 (5)	0.2785 (4)	0.4772 (4)	0.0655 (12)	
N5	−0.0008 (4)	0.1692 (4)	0.4422 (4)	0.0550 (11)	
C1	0.3678 (5)	0.0384 (4)	0.5593 (4)	0.0477 (12)	
C2	0.4117 (6)	−0.0673 (6)	0.5232 (5)	0.0692 (17)	
H2	0.3791	−0.0924	0.4506	0.083*	
C3	0.5033 (6)	−0.1362 (5)	0.5932 (5)	0.0659 (16)	
H3	0.5325	−0.2075	0.5685	0.079*	
C4	0.5503 (5)	−0.0981 (5)	0.6990 (5)	0.0521 (13)	
C5	0.5094 (4)	0.0081 (4)	0.7364 (4)	0.0440 (12)	
H5	0.5448	0.0330	0.8085	0.053*	
C6	0.4170 (4)	0.0775 (4)	0.6685 (4)	0.0400 (11)	
C7	0.3603 (4)	0.1881 (4)	0.7103 (4)	0.0387 (11)	
H7	0.3533	0.2471	0.6509	0.046*	
C8	0.5485 (5)	0.3221 (6)	0.7870 (4)	0.0616 (15)	
H8A	0.5598	0.3183	0.7093	0.074*	
H8B	0.6365	0.3074	0.8338	0.074*	
C9	0.4947 (6)	0.4396 (6)	0.8129 (6)	0.0737 (18)	
H9A	0.4350	0.4718	0.7483	0.088*	
H9B	0.5689	0.4941	0.8371	0.088*	
C10	0.4158 (6)	0.4135 (5)	0.9067 (5)	0.0657 (16)	
H10A	0.3404	0.4679	0.9054	0.079*	
H10B	0.4752	0.4184	0.9789	0.079*	
C11	0.3630 (4)	0.2884 (4)	0.8834 (4)	0.0458 (12)	
H11	0.3793	0.2453	0.9543	0.055*	
C12	0.2136 (5)	0.2693 (4)	0.8294 (4)	0.0460 (12)	
H12	0.1619	0.2414	0.8861	0.055*	
C13	0.2173 (5)	0.1730 (4)	0.7424 (4)	0.0389 (11)	
H13	0.1475	0.1897	0.6767	0.047*	
C14	0.1887 (4)	0.0524 (4)	0.7888 (4)	0.0423 (11)	
H14	0.2418	−0.0068	0.7562	0.051*	
C15	0.1610 (6)	−0.0349 (5)	0.9550 (4)	0.0501 (14)	
H15	0.1313	−0.0070	1.0234	0.060*	
C16	0.1832 (6)	−0.2298 (4)	0.9097 (5)	0.0566 (14)	
C17	0.2849 (7)	−0.2733 (7)	0.8436 (7)	0.101 (3)	
H17A	0.2484	−0.3402	0.8007	0.152*	
H17B	0.3672	−0.2952	0.8932	0.152*	
H17C	0.3052	−0.2127	0.7942	0.152*	
C18	0.1389 (8)	−0.3214 (6)	0.9836 (6)	0.087 (2)	
H18A	0.0723	−0.2889	1.0237	0.130*	
H18B	0.2168	−0.3482	1.0357	0.130*	
H18C	0.0992	−0.3861	0.9389	0.130*	
C19	0.0388 (5)	−0.0700 (4)	0.8692 (4)	0.0482 (13)	
H19	−0.0472	−0.0670	0.8986	0.058*	
C20	0.0399 (4)	0.0145 (4)	0.7713 (4)	0.0429 (11)	
H20	0.0154	−0.0269	0.6999	0.051*	
C21	−0.1598 (5)	0.1165 (6)	0.6832 (5)	0.0614 (15)	
H21A	−0.2284	0.1712	0.6997	0.074*	
H21B	−0.2028	0.0403	0.6694	0.074*	
C22	−0.1072 (5)	0.1562 (5)	0.5805 (5)	0.0551 (14)	
C23	−0.0554 (5)	0.0906 (5)	0.5042 (4)	0.0523 (13)	
C24	0.0826 (6)	0.1482 (5)	0.3571 (4)	0.0568 (14)	
H24A	0.1209	0.2219	0.3373	0.068*	
H24B	0.0252	0.1173	0.2906	0.068*	
C25	0.1960 (6)	0.0642 (5)	0.3954 (4)	0.0567 (14)	
H25A	0.1591	−0.0121	0.4091	0.068*	
H25B	0.2533	0.0557	0.3387	0.068*	

### (I) (Z)-15-Bromo-32,32-dimethyl-21-nitro-22,23,25,26,27,27a,33a,35,36,36a-decahydro-21H,61H-4,9-dioxa-2(3,2)-pyrrolizina-6(4,1)-triazola-3(5,6)-furo[2,3-d][1,3]dioxola-1(1,2)-benzenacyclononaphane Atomic displacement parameters (Å^2^)

**Table:**

	*U*^11^	*U*^22^	*U*^33^	*U*^12^	*U*^13^	*U*^23^
Br1	0.0711 (3)	0.0748 (4)	0.0813 (4)	0.0282 (4)	0.0100 (3)	0.0041 (4)
O1	0.107 (3)	0.052 (3)	0.077 (3)	0.006 (2)	−0.011 (3)	0.013 (2)
O2	0.105 (3)	0.074 (3)	0.115 (4)	0.041 (3)	0.018 (3)	−0.016 (3)
O3	0.071 (2)	0.050 (2)	0.054 (2)	−0.015 (2)	−0.0156 (18)	0.0142 (19)
O4	0.098 (3)	0.046 (2)	0.096 (4)	−0.009 (2)	−0.047 (3)	0.010 (2)
O5	0.078 (2)	0.046 (2)	0.063 (2)	−0.002 (2)	−0.0130 (18)	−0.006 (2)
O6	0.0474 (18)	0.058 (2)	0.051 (2)	0.0146 (18)	0.0077 (16)	−0.0018 (17)
O7	0.077 (2)	0.046 (2)	0.041 (2)	0.0036 (19)	−0.0054 (18)	−0.0087 (17)
N1	0.047 (2)	0.046 (2)	0.040 (2)	−0.001 (2)	0.0014 (19)	−0.0033 (19)
N2	0.071 (3)	0.044 (3)	0.071 (4)	0.010 (3)	−0.005 (3)	−0.009 (3)
N3	0.082 (3)	0.046 (3)	0.063 (3)	0.018 (2)	−0.008 (2)	−0.002 (2)
N4	0.094 (3)	0.036 (3)	0.061 (3)	0.007 (2)	−0.002 (3)	0.004 (2)
N5	0.065 (3)	0.049 (3)	0.045 (3)	0.002 (2)	−0.007 (2)	−0.005 (2)
C1	0.056 (3)	0.046 (3)	0.040 (3)	0.001 (3)	0.008 (2)	−0.003 (2)
C2	0.088 (4)	0.068 (4)	0.050 (4)	0.015 (4)	0.008 (3)	−0.018 (3)
C3	0.077 (4)	0.055 (3)	0.067 (4)	0.021 (3)	0.017 (3)	−0.013 (3)
C4	0.048 (3)	0.051 (3)	0.059 (4)	0.005 (3)	0.015 (3)	0.001 (3)
C5	0.041 (2)	0.045 (3)	0.048 (3)	0.000 (2)	0.013 (2)	−0.006 (2)
C6	0.041 (2)	0.043 (3)	0.039 (3)	−0.002 (2)	0.014 (2)	−0.003 (2)
C7	0.049 (3)	0.035 (2)	0.031 (2)	−0.005 (2)	0.005 (2)	−0.004 (2)
C8	0.057 (3)	0.073 (4)	0.052 (3)	−0.018 (3)	0.001 (2)	−0.011 (3)
C9	0.075 (4)	0.054 (4)	0.089 (5)	−0.017 (3)	0.003 (4)	0.006 (3)
C10	0.074 (4)	0.043 (3)	0.074 (4)	−0.002 (3)	−0.003 (3)	−0.022 (3)
C11	0.057 (3)	0.041 (3)	0.038 (2)	0.003 (3)	0.007 (2)	0.002 (2)
C12	0.058 (3)	0.035 (3)	0.046 (3)	0.006 (2)	0.012 (2)	−0.001 (2)
C13	0.048 (3)	0.031 (2)	0.037 (3)	0.003 (2)	0.003 (2)	−0.002 (2)
C14	0.046 (2)	0.039 (3)	0.041 (3)	0.001 (2)	0.004 (2)	0.002 (2)
C15	0.068 (3)	0.048 (3)	0.035 (3)	−0.007 (3)	0.011 (3)	−0.004 (2)
C16	0.068 (3)	0.041 (3)	0.057 (3)	−0.008 (3)	−0.001 (3)	0.012 (3)
C17	0.093 (5)	0.072 (5)	0.149 (8)	0.017 (4)	0.051 (5)	0.031 (5)
C18	0.135 (6)	0.062 (4)	0.064 (4)	−0.021 (4)	0.018 (4)	0.013 (4)
C19	0.050 (3)	0.046 (3)	0.049 (3)	−0.008 (3)	0.009 (2)	−0.001 (3)
C20	0.045 (3)	0.045 (3)	0.040 (3)	0.004 (2)	0.010 (2)	−0.002 (2)
C21	0.043 (3)	0.077 (4)	0.063 (4)	0.015 (3)	0.006 (3)	−0.003 (3)
C22	0.048 (3)	0.061 (4)	0.051 (3)	0.010 (3)	−0.007 (2)	−0.001 (3)
C23	0.058 (3)	0.056 (3)	0.039 (3)	−0.006 (3)	−0.003 (2)	0.004 (3)
C24	0.082 (4)	0.053 (3)	0.032 (3)	−0.008 (3)	−0.001 (3)	0.005 (2)
C25	0.080 (3)	0.056 (3)	0.033 (3)	−0.012 (3)	0.007 (3)	−0.008 (3)

### (I) (Z)-15-Bromo-32,32-dimethyl-21-nitro-22,23,25,26,27,27a,33a,35,36,36a-decahydro-21H,61H-4,9-dioxa-2(3,2)-pyrrolizina-6(4,1)-triazola-3(5,6)-furo[2,3-d][1,3]dioxola-1(1,2)-benzenacyclononaphane Geometric parameters (Å, º)

**Table:**

Br1—C4	1.908 (5)	C9—C10	1.519 (8)
O1—N2	1.214 (6)	C9—H9A	0.9700
O2—N2	1.218 (6)	C9—H9B	0.9700
O3—C15	1.385 (6)	C10—C11	1.530 (8)
O3—C14	1.454 (5)	C10—H10A	0.9700
O4—C15	1.372 (7)	C10—H10B	0.9700
O4—C16	1.414 (7)	C11—C12	1.529 (7)
O5—C19	1.405 (6)	C11—H11	0.9800
O5—C16	1.408 (6)	C12—C13	1.530 (7)
O6—C20	1.415 (6)	C12—H12	0.9800
O6—C21	1.433 (6)	C13—C14	1.533 (7)
O7—C1	1.362 (6)	C13—H13	0.9800
O7—C25	1.432 (6)	C14—C20	1.516 (6)
N1—C7	1.458 (6)	C14—H14	0.9800
N1—C11	1.459 (6)	C15—C19	1.510 (7)
N1—C8	1.469 (6)	C15—H15	0.9800
N2—C12	1.490 (7)	C16—C17	1.478 (8)
N3—N4	1.319 (6)	C16—C18	1.492 (8)
N3—C22	1.358 (7)	C17—H17A	0.9600
N4—N5	1.337 (6)	C17—H17B	0.9600
N5—C23	1.344 (6)	C17—H17C	0.9600
N5—C24	1.450 (6)	C18—H18A	0.9600
C1—C2	1.380 (8)	C18—H18B	0.9600
C1—C6	1.405 (7)	C18—H18C	0.9600
C2—C3	1.378 (8)	C19—C20	1.533 (7)
C2—H2	0.9300	C19—H19	0.9800
C3—C4	1.361 (8)	C20—H20	0.9800
C3—H3	0.9300	C21—C22	1.501 (8)
C4—C5	1.380 (7)	C21—H21A	0.9700
C5—C6	1.373 (7)	C21—H21B	0.9700
C5—H5	0.9300	C22—C23	1.359 (7)
C6—C7	1.506 (6)	C24—C25	1.490 (8)
C7—C13	1.542 (6)	C24—H24A	0.9700
C7—H7	0.9800	C24—H24B	0.9700
C8—C9	1.497 (9)	C25—H25A	0.9700
C8—H8A	0.9700	C25—H25B	0.9700
C8—H8B	0.9700		
			
C15—O3—C14	108.8 (4)	C12—C13—C14	111.3 (4)
C15—O4—C16	112.1 (4)	C12—C13—C7	103.1 (4)
C19—O5—C16	111.0 (4)	C14—C13—C7	115.5 (4)
C20—O6—C21	113.8 (4)	C12—C13—H13	108.9
C1—O7—C25	118.7 (4)	C14—C13—H13	108.9
C7—N1—C11	110.0 (3)	C7—C13—H13	108.9
C7—N1—C8	114.9 (4)	O3—C14—C20	104.4 (3)
C11—N1—C8	108.4 (4)	O3—C14—C13	109.3 (4)
O1—N2—O2	123.5 (6)	C20—C14—C13	116.2 (4)
O1—N2—C12	118.5 (5)	O3—C14—H14	108.9
O2—N2—C12	118.0 (5)	C20—C14—H14	108.9
N4—N3—C22	108.2 (4)	C13—C14—H14	108.9
N3—N4—N5	107.1 (4)	O4—C15—O3	111.4 (4)
N4—N5—C23	111.2 (4)	O4—C15—C19	105.7 (4)
N4—N5—C24	119.7 (5)	O3—C15—C19	108.3 (4)
C23—N5—C24	128.6 (4)	O4—C15—H15	110.4
O7—C1—C2	124.4 (5)	O3—C15—H15	110.4
O7—C1—C6	115.6 (4)	C19—C15—H15	110.4
C2—C1—C6	120.1 (5)	O5—C16—O4	105.2 (4)
C3—C2—C1	120.8 (5)	O5—C16—C17	109.1 (5)
C3—C2—H2	119.6	O4—C16—C17	109.5 (5)
C1—C2—H2	119.6	O5—C16—C18	110.9 (5)
C4—C3—C2	118.9 (5)	O4—C16—C18	108.8 (5)
C4—C3—H3	120.6	C17—C16—C18	113.0 (5)
C2—C3—H3	120.6	C16—C17—H17A	109.5
C3—C4—C5	121.3 (5)	C16—C17—H17B	109.5
C3—C4—Br1	119.7 (4)	H17A—C17—H17B	109.5
C5—C4—Br1	119.0 (4)	C16—C17—H17C	109.5
C6—C5—C4	120.8 (5)	H17A—C17—H17C	109.5
C6—C5—H5	119.6	H17B—C17—H17C	109.5
C4—C5—H5	119.6	C16—C18—H18A	109.5
C5—C6—C1	118.1 (4)	C16—C18—H18B	109.5
C5—C6—C7	122.0 (4)	H18A—C18—H18B	109.5
C1—C6—C7	119.7 (4)	C16—C18—H18C	109.5
N1—C7—C6	112.7 (4)	H18A—C18—H18C	109.5
N1—C7—C13	105.7 (3)	H18B—C18—H18C	109.5
C6—C7—C13	113.8 (4)	O5—C19—C15	104.5 (4)
N1—C7—H7	108.1	O5—C19—C20	109.3 (4)
C6—C7—H7	108.1	C15—C19—C20	104.9 (4)
C13—C7—H7	108.1	O5—C19—H19	112.5
N1—C8—C9	106.6 (4)	C15—C19—H19	112.5
N1—C8—H8A	110.4	C20—C19—H19	112.5
C9—C8—H8A	110.4	O6—C20—C14	112.8 (4)
N1—C8—H8B	110.4	O6—C20—C19	110.5 (3)
C9—C8—H8B	110.4	C14—C20—C19	102.0 (4)
H8A—C8—H8B	108.6	O6—C20—H20	110.4
C8—C9—C10	103.3 (5)	C14—C20—H20	110.4
C8—C9—H9A	111.1	C19—C20—H20	110.4
C10—C9—H9A	111.1	O6—C21—C22	111.0 (4)
C8—C9—H9B	111.1	O6—C21—H21A	109.4
C10—C9—H9B	111.1	C22—C21—H21A	109.4
H9A—C9—H9B	109.1	O6—C21—H21B	109.4
C9—C10—C11	104.4 (4)	C22—C21—H21B	109.4
C9—C10—H10A	110.9	H21A—C21—H21B	108.0
C11—C10—H10A	110.9	N3—C22—C23	109.0 (5)
C9—C10—H10B	110.9	N3—C22—C21	121.5 (5)
C11—C10—H10B	110.9	C23—C22—C21	128.7 (5)
H10A—C10—H10B	108.9	N5—C23—C22	104.5 (5)
N1—C11—C12	106.9 (4)	N5—C24—C25	112.1 (4)
N1—C11—C10	106.7 (4)	N5—C24—H24A	109.2
C12—C11—C10	119.2 (4)	C25—C24—H24A	109.2
N1—C11—H11	107.9	N5—C24—H24B	109.2
C12—C11—H11	107.9	C25—C24—H24B	109.2
C10—C11—H11	107.9	H24A—C24—H24B	107.9
N2—C12—C11	112.9 (4)	O7—C25—C24	107.7 (4)
N2—C12—C13	111.0 (4)	O7—C25—H25A	110.2
C11—C12—C13	105.2 (4)	C24—C25—H25A	110.2
N2—C12—H12	109.2	O7—C25—H25B	110.2
C11—C12—H12	109.2	C24—C25—H25B	110.2
C13—C12—H12	109.2	H25A—C25—H25B	108.5
			
C22—N3—N4—N5	0.7 (6)	C6—C7—C13—C12	153.8 (4)
N3—N4—N5—C23	−0.4 (6)	N1—C7—C13—C14	−92.1 (5)
N3—N4—N5—C24	−173.2 (4)	C6—C7—C13—C14	32.2 (5)
C25—O7—C1—C2	13.2 (7)	C15—O3—C14—C20	32.4 (5)
C25—O7—C1—C6	−165.4 (4)	C15—O3—C14—C13	157.4 (4)
O7—C1—C2—C3	−178.0 (5)	C12—C13—C14—O3	−27.6 (5)
C6—C1—C2—C3	0.4 (8)	C7—C13—C14—O3	89.5 (4)
C1—C2—C3—C4	−0.2 (9)	C12—C13—C14—C20	90.2 (5)
C2—C3—C4—C5	−0.8 (8)	C7—C13—C14—C20	−152.8 (4)
C2—C3—C4—Br1	178.5 (4)	C16—O4—C15—O3	−114.9 (5)
C3—C4—C5—C6	1.6 (7)	C16—O4—C15—C19	2.5 (6)
Br1—C4—C5—C6	−177.7 (3)	C14—O3—C15—O4	98.4 (5)
C4—C5—C6—C1	−1.3 (6)	C14—O3—C15—C19	−17.4 (5)
C4—C5—C6—C7	174.1 (4)	C19—O5—C16—O4	−11.1 (5)
O7—C1—C6—C5	178.9 (4)	C19—O5—C16—C17	−128.5 (5)
C2—C1—C6—C5	0.3 (7)	C19—O5—C16—C18	106.4 (5)
O7—C1—C6—C7	3.4 (6)	C15—O4—C16—O5	4.9 (6)
C2—C1—C6—C7	−175.2 (4)	C15—O4—C16—C17	122.1 (5)
C11—N1—C7—C6	−145.0 (4)	C15—O4—C16—C18	−114.0 (5)
C8—N1—C7—C6	92.3 (5)	C16—O5—C19—C15	12.4 (5)
C11—N1—C7—C13	−20.1 (5)	C16—O5—C19—C20	124.2 (4)
C8—N1—C7—C13	−142.8 (4)	O4—C15—C19—O5	−8.9 (5)
C5—C6—C7—N1	21.8 (6)	O3—C15—C19—O5	110.5 (4)
C1—C6—C7—N1	−162.9 (4)	O4—C15—C19—C20	−123.9 (4)
C5—C6—C7—C13	−98.6 (5)	O3—C15—C19—C20	−4.4 (5)
C1—C6—C7—C13	76.7 (5)	C21—O6—C20—C14	128.0 (4)
C7—N1—C8—C9	103.5 (5)	C21—O6—C20—C19	−118.6 (4)
C11—N1—C8—C9	−20.0 (5)	O3—C14—C20—O6	85.4 (4)
N1—C8—C9—C10	31.6 (6)	C13—C14—C20—O6	−35.0 (5)
C8—C9—C10—C11	−30.9 (6)	O3—C14—C20—C19	−33.1 (5)
C7—N1—C11—C12	2.2 (5)	C13—C14—C20—C19	−153.5 (4)
C8—N1—C11—C12	128.6 (4)	O5—C19—C20—O6	151.2 (4)
C7—N1—C11—C10	−126.4 (4)	C15—C19—C20—O6	−97.2 (4)
C8—N1—C11—C10	0.0 (5)	O5—C19—C20—C14	−88.6 (4)
C9—C10—C11—N1	19.4 (6)	C15—C19—C20—C14	22.9 (5)
C9—C10—C11—C12	−101.6 (5)	C20—O6—C21—C22	−71.5 (6)
O1—N2—C12—C11	70.1 (6)	N4—N3—C22—C23	−0.8 (6)
O2—N2—C12—C11	−108.9 (5)	N4—N3—C22—C21	169.5 (4)
O1—N2—C12—C13	−47.7 (6)	O6—C21—C22—N3	−81.0 (6)
O2—N2—C12—C13	133.3 (5)	O6—C21—C22—C23	87.3 (7)
N1—C11—C12—N2	−104.5 (5)	N4—N5—C23—C22	−0.1 (6)
C10—C11—C12—N2	16.4 (6)	C24—N5—C23—C22	172.0 (5)
N1—C11—C12—C13	16.7 (5)	N3—C22—C23—N5	0.5 (6)
C10—C11—C12—C13	137.6 (4)	C21—C22—C23—N5	−168.9 (5)
N2—C12—C13—C14	−141.1 (4)	N4—N5—C24—C25	123.3 (5)
C11—C12—C13—C14	96.5 (4)	C23—N5—C24—C25	−48.1 (7)
N2—C12—C13—C7	94.6 (5)	C1—O7—C25—C24	167.4 (4)
C11—C12—C13—C7	−27.9 (5)	N5—C24—C25—O7	−56.2 (6)
N1—C7—C13—C12	29.5 (5)		

### (I) (Z)-15-Bromo-32,32-dimethyl-21-nitro-22,23,25,26,27,27a,33a,35,36,36a-decahydro-21H,61H-4,9-dioxa-2(3,2)-pyrrolizina-6(4,1)-triazola-3(5,6)-furo[2,3-d][1,3]dioxola-1(1,2)-benzenacyclononaphane Hydrogen-bond geometry (Å, º)

**Table:**

*D*—H···*A*	*D*—H	H···*A*	*D*···*A*	*D*—H···*A*
C8—H8*B*···O4^i^	0.97	2.51	3.295 (7)	138
C18—H18*C*···O2^ii^	0.96	2.57	3.509 (9)	164
C25—H25*A*···N3^iii^	0.97	2.62	3.589 (7)	173

Symmetry codes: (i) −*x*+1, *y*+1/2, −*z*+2; (ii) *x*, *y*−1, *z*; (iii) −*x*, *y*−1/2, −*z*+1.

### (II) (Z)-32,32-Dimethyl-27-nitro-25,26,27,27a,33a,35,36,36a-octahydro-21H,23H,61H-4,9-dioxa-2(5,6)-pyrrolo[1,2-c]thiazola-6(4,1)-triazola-3(5,6)-furo[2,3-d][1,3]dioxola-1(1,2)-benzenacyclononaphane Crystal data

**Table:**

C_24_H_29_N_5_O_7_S	*F*(000) = 560
*M**_r_* = 531.58	*D*_x_ = 1.401 Mg m^−^^3^
Monoclinic, *P*2_1_	Mo *K*α radiation, λ = 0.71073 Å
*a* = 8.756 (5) Å	Cell parameters from 4712 reflections
*b* = 10.811 (5) Å	θ = 1.5–22.3°
*c* = 13.569 (5) Å	µ = 0.18 mm^−^^1^
β = 101.122 (5)°	*T* = 293 K
*V* = 1260.3 (10) Å^3^	Block, colourless
*Z* = 2	0.20 × 0.15 × 0.10 mm

### (II) (Z)-32,32-Dimethyl-27-nitro-25,26,27,27a,33a,35,36,36a-octahydro-21H,23H,61H-4,9-dioxa-2(5,6)-pyrrolo[1,2-c]thiazola-6(4,1)-triazola-3(5,6)-furo[2,3-d][1,3]dioxola-1(1,2)-benzenacyclononaphane Data collection

**Table:**

Bruker SMART APEXII area-detector diffractometer	4712 independent reflections
Radiation source: fine-focus sealed tube	2862 reflections with *I* > 2σ(*I*)
Graphite monochromator	*R*_int_ = 0.041
ω and φ scans	θ_max_ = 28.3°, θ_min_ = 1.5°
Absorption correction: multi-scan (*SADABS*; Bruker, 2008)	*h* = −11→11
*T*_min_ = 0.964, *T*_max_ = 0.982	*k* = −13→14
11813 measured reflections	*l* = −17→18

### (II) (Z)-32,32-Dimethyl-27-nitro-25,26,27,27a,33a,35,36,36a-octahydro-21H,23H,61H-4,9-dioxa-2(5,6)-pyrrolo[1,2-c]thiazola-6(4,1)-triazola-3(5,6)-furo[2,3-d][1,3]dioxola-1(1,2)-benzenacyclononaphane Refinement

**Table:**

Refinement on *F*^2^	Hydrogen site location: inferred from neighbouring sites
Least-squares matrix: full	H-atom parameters constrained
*R*[*F*^2^ > 2σ(*F*^2^)] = 0.046	*w* = 1/[σ^2^(*F*_o_^2^) + (0.0413*P*)^2^] where *P* = (*F*_o_^2^ + 2*F*_c_^2^)/3
*wR*(*F*^2^) = 0.103	(Δ/σ)_max_ < 0.001
*S* = 1.00	Δρ_max_ = 0.17 e Å^−^^3^
4712 reflections	Δρ_min_ = −0.24 e Å^−^^3^
336 parameters	Absolute structure: Flack *x* determined using 794 quotients [(*I*^+^)-(*I*^-^)]/[(*I*^+^)+(*I*^-^)] (Parsons *et al.*, 2013)
1 restraint	Absolute structure parameter: −0.10 (9)

### (II) (Z)-32,32-Dimethyl-27-nitro-25,26,27,27a,33a,35,36,36a-octahydro-21H,23H,61H-4,9-dioxa-2(5,6)-pyrrolo[1,2-c]thiazola-6(4,1)-triazola-3(5,6)-furo[2,3-d][1,3]dioxola-1(1,2)-benzenacyclononaphane Special details

**Table:**

Geometry. All esds (except the esd in the dihedral angle between two l.s. planes) are estimated using the full covariance matrix. The cell esds are taken into account individually in the estimation of esds in distances, angles and torsion angles; correlations between esds in cell parameters are only used when they are defined by crystal symmetry. An approximate (isotropic) treatment of cell esds is used for estimating esds involving l.s. planes.

### (II) (Z)-32,32-Dimethyl-27-nitro-25,26,27,27a,33a,35,36,36a-octahydro-21H,23H,61H-4,9-dioxa-2(5,6)-pyrrolo[1,2-c]thiazola-6(4,1)-triazola-3(5,6)-furo[2,3-d][1,3]dioxola-1(1,2)-benzenacyclononaphane Fractional atomic coordinates and isotropic or equivalent isotropic displacement parameters (Å^2^)

**Table:**

	*x*	*y*	*z*	*U*_iso_*/*U*_eq_	
S9	0.85963 (16)	−0.08380 (12)	0.26658 (11)	0.0856 (5)	
O1	0.2482 (4)	−0.0495 (3)	0.0593 (3)	0.0996 (13)	
O2	0.2983 (4)	−0.0548 (3)	0.2195 (3)	0.0873 (11)	
O3	0.2622 (3)	0.2632 (2)	0.11689 (17)	0.0547 (7)	
O4	0.2164 (3)	0.4638 (3)	0.06070 (18)	0.0578 (7)	
O5	0.2374 (4)	0.5327 (2)	0.2191 (2)	0.0631 (8)	
O6	0.2153 (3)	0.2254 (2)	0.31708 (18)	0.0496 (7)	
O7	0.8007 (3)	0.2715 (2)	0.43718 (18)	0.0538 (7)	
N1	0.7152 (3)	0.1098 (3)	0.1692 (2)	0.0434 (8)	
N2	0.3210 (4)	−0.0163 (3)	0.1400 (3)	0.0598 (10)	
N3	0.4307 (4)	0.0753 (3)	0.4787 (3)	0.0597 (9)	
N4	0.5760 (4)	0.0738 (3)	0.5266 (3)	0.0600 (10)	
N5	0.6117 (4)	0.1899 (3)	0.5601 (2)	0.0521 (9)	
C1	0.7925 (4)	0.3522 (4)	0.3582 (3)	0.0441 (9)	
C2	0.8434 (5)	0.4733 (4)	0.3693 (3)	0.0553 (11)	
H2	0.8865	0.5038	0.4326	0.066*	
C3	0.8302 (5)	0.5485 (4)	0.2866 (4)	0.0615 (12)	
H3	0.8615	0.6307	0.2946	0.074*	
C4	0.7715 (4)	0.5040 (4)	0.1926 (4)	0.0558 (12)	
H4	0.7642	0.5551	0.1368	0.067*	
C5	0.7234 (4)	0.3827 (4)	0.1817 (3)	0.0512 (10)	
H5	0.6863	0.3518	0.1176	0.061*	
C6	0.7288 (4)	0.3054 (4)	0.2638 (3)	0.0400 (9)	
C7	0.6631 (4)	0.1762 (3)	0.2522 (3)	0.0404 (9)	
H7	0.7020	0.1310	0.3146	0.049*	
C8	0.8712 (5)	0.0606 (4)	0.1945 (3)	0.0629 (12)	
H8B	0.9400	0.1196	0.2348	0.076*	
H8A	0.9110	0.0431	0.1340	0.076*	
C10	0.6519 (5)	−0.0954 (4)	0.2256 (3)	0.0644 (12)	
H10A	0.6232	−0.1768	0.1980	0.077*	
H10B	0.5990	−0.0806	0.2811	0.077*	
C11	0.6090 (4)	0.0046 (4)	0.1445 (3)	0.0458 (10)	
H11	0.6163	−0.0289	0.0784	0.055*	
C12	0.4536 (4)	0.0711 (3)	0.1392 (3)	0.0405 (9)	
H12	0.4314	0.1185	0.0765	0.049*	
C13	0.4804 (4)	0.1625 (3)	0.2271 (2)	0.0359 (8)	
H13	0.4481	0.1222	0.2844	0.043*	
C14	0.3855 (4)	0.2812 (3)	0.2042 (2)	0.0377 (8)	
H14	0.4542	0.3478	0.1900	0.045*	
C15	0.1637 (5)	0.3661 (3)	0.1128 (3)	0.0474 (10)	
H15	0.0560	0.3442	0.0832	0.057*	
C16	0.2264 (5)	0.5744 (4)	0.1192 (3)	0.0458 (10)	
C17	0.3722 (5)	0.6412 (5)	0.1111 (4)	0.0824 (16)	
H17A	0.4588	0.5852	0.1255	0.124*	
H17B	0.3881	0.7083	0.1582	0.124*	
H17C	0.3638	0.6731	0.0442	0.124*	
C18	0.0832 (5)	0.6512 (4)	0.0852 (3)	0.0667 (13)	
H18C	0.0792	0.6775	0.0171	0.100*	
H18B	0.0864	0.7224	0.1278	0.100*	
H18A	−0.0075	0.6029	0.0888	0.100*	
C19	0.1766 (4)	0.4125 (4)	0.2205 (3)	0.0453 (9)	
H19	0.0763	0.4113	0.2423	0.054*	
C20	0.2947 (4)	0.3260 (3)	0.2824 (3)	0.0398 (9)	
H20	0.3617	0.3694	0.3379	0.048*	
C21	0.2186 (5)	0.2264 (5)	0.4235 (3)	0.0630 (12)	
H21A	0.1419	0.1685	0.4389	0.076*	
H21B	0.1911	0.3083	0.4436	0.076*	
C22	0.3746 (5)	0.1924 (4)	0.4809 (3)	0.0504 (10)	
C23	0.4913 (5)	0.2649 (4)	0.5322 (3)	0.0548 (11)	
H23	0.4870	0.3492	0.5449	0.066*	
C24	0.7723 (5)	0.2219 (4)	0.6012 (3)	0.0624 (12)	
H24A	0.8373	0.1489	0.6036	0.075*	
H24B	0.7806	0.2527	0.6692	0.075*	
C25	0.8274 (5)	0.3194 (4)	0.5369 (3)	0.0585 (11)	
H25A	0.7699	0.3957	0.5393	0.070*	
H25B	0.9373	0.3361	0.5603	0.070*	

### (II) (Z)-32,32-Dimethyl-27-nitro-25,26,27,27a,33a,35,36,36a-octahydro-21H,23H,61H-4,9-dioxa-2(5,6)-pyrrolo[1,2-c]thiazola-6(4,1)-triazola-3(5,6)-furo[2,3-d][1,3]dioxola-1(1,2)-benzenacyclononaphane Atomic displacement parameters (Å^2^)

**Table:**

	*U*^11^	*U*^22^	*U*^33^	*U*^12^	*U*^13^	*U*^23^
S9	0.0720 (8)	0.0642 (8)	0.1050 (10)	0.0174 (7)	−0.0215 (7)	0.0085 (8)
O1	0.097 (3)	0.087 (3)	0.096 (3)	−0.032 (2)	−0.028 (2)	−0.016 (2)
O2	0.104 (3)	0.072 (3)	0.095 (3)	−0.032 (2)	0.042 (2)	−0.011 (2)
O3	0.0668 (19)	0.0457 (16)	0.0421 (15)	0.0084 (15)	−0.0129 (13)	−0.0093 (13)
O4	0.085 (2)	0.0445 (16)	0.0423 (15)	0.0019 (16)	0.0087 (14)	0.0044 (14)
O5	0.107 (2)	0.0362 (15)	0.0420 (17)	0.0049 (16)	0.0050 (16)	0.0013 (13)
O6	0.0474 (15)	0.0523 (17)	0.0492 (16)	−0.0038 (13)	0.0098 (12)	0.0093 (13)
O7	0.0715 (19)	0.0501 (17)	0.0370 (14)	−0.0061 (15)	0.0041 (12)	−0.0081 (13)
N1	0.0393 (18)	0.0464 (19)	0.0439 (18)	0.0011 (15)	0.0064 (14)	−0.0083 (15)
N2	0.063 (2)	0.040 (2)	0.075 (3)	−0.001 (2)	0.008 (2)	−0.010 (2)
N3	0.068 (3)	0.048 (2)	0.059 (2)	0.000 (2)	0.0015 (19)	0.0108 (18)
N4	0.075 (3)	0.039 (2)	0.059 (2)	−0.0001 (19)	−0.004 (2)	0.0038 (18)
N5	0.066 (2)	0.046 (2)	0.0411 (19)	0.0018 (19)	0.0010 (16)	0.0059 (16)
C1	0.040 (2)	0.042 (2)	0.049 (2)	−0.0014 (18)	0.0069 (18)	−0.002 (2)
C2	0.054 (3)	0.052 (3)	0.061 (3)	−0.006 (2)	0.012 (2)	−0.010 (2)
C3	0.049 (3)	0.041 (2)	0.096 (4)	−0.005 (2)	0.020 (3)	0.000 (3)
C4	0.039 (2)	0.053 (3)	0.073 (3)	−0.003 (2)	0.004 (2)	0.020 (2)
C5	0.044 (2)	0.050 (3)	0.056 (3)	−0.003 (2)	−0.0004 (19)	0.007 (2)
C6	0.035 (2)	0.040 (2)	0.044 (2)	0.0013 (17)	0.0037 (16)	0.0001 (18)
C7	0.042 (2)	0.040 (2)	0.037 (2)	0.0005 (18)	0.0015 (16)	−0.0002 (17)
C8	0.044 (2)	0.067 (3)	0.076 (3)	0.012 (2)	0.005 (2)	−0.008 (3)
C10	0.069 (3)	0.046 (2)	0.074 (3)	0.008 (2)	0.002 (2)	0.005 (2)
C11	0.045 (2)	0.049 (2)	0.039 (2)	0.0041 (19)	−0.0012 (17)	−0.0080 (18)
C12	0.045 (2)	0.036 (2)	0.040 (2)	−0.0010 (18)	0.0073 (16)	−0.0034 (17)
C13	0.040 (2)	0.035 (2)	0.0319 (19)	0.0008 (17)	0.0051 (15)	0.0013 (16)
C14	0.039 (2)	0.038 (2)	0.0340 (19)	−0.0019 (17)	0.0022 (15)	−0.0017 (16)
C15	0.044 (2)	0.038 (2)	0.053 (3)	0.0008 (19)	−0.0082 (18)	0.0029 (19)
C16	0.052 (2)	0.037 (2)	0.046 (2)	−0.001 (2)	0.0051 (18)	0.0005 (19)
C17	0.056 (3)	0.068 (3)	0.124 (4)	−0.008 (3)	0.019 (3)	0.008 (3)
C18	0.061 (3)	0.059 (3)	0.080 (3)	0.015 (2)	0.012 (2)	0.016 (3)
C19	0.044 (2)	0.044 (2)	0.050 (2)	0.005 (2)	0.0130 (18)	0.005 (2)
C20	0.042 (2)	0.035 (2)	0.042 (2)	−0.0008 (18)	0.0079 (16)	0.0038 (18)
C21	0.060 (3)	0.077 (3)	0.057 (3)	0.008 (3)	0.025 (2)	0.018 (3)
C22	0.055 (3)	0.053 (3)	0.045 (2)	0.006 (2)	0.014 (2)	0.014 (2)
C23	0.072 (3)	0.044 (2)	0.051 (2)	0.008 (2)	0.017 (2)	0.006 (2)
C24	0.073 (3)	0.072 (3)	0.038 (2)	−0.006 (3)	0.000 (2)	0.001 (2)
C25	0.061 (3)	0.069 (3)	0.042 (2)	−0.009 (2)	0.0005 (19)	−0.014 (2)

### (II) (Z)-32,32-Dimethyl-27-nitro-25,26,27,27a,33a,35,36,36a-octahydro-21H,23H,61H-4,9-dioxa-2(5,6)-pyrrolo[1,2-c]thiazola-6(4,1)-triazola-3(5,6)-furo[2,3-d][1,3]dioxola-1(1,2)-benzenacyclononaphane Geometric parameters (Å, º)

**Table:**

S9—C10	1.801 (4)	C8—H8B	0.9700
S9—C8	1.855 (5)	C8—H8A	0.9700
O1—N2	1.210 (4)	C10—C11	1.536 (6)
O2—N2	1.207 (4)	C10—H10A	0.9700
O3—C15	1.402 (4)	C10—H10B	0.9700
O3—C14	1.454 (4)	C11—C12	1.528 (5)
O4—C15	1.398 (5)	C11—H11	0.9800
O4—C16	1.428 (5)	C12—C13	1.532 (5)
O5—C19	1.406 (5)	C12—H12	0.9800
O5—C16	1.415 (4)	C13—C14	1.528 (5)
O6—C20	1.418 (4)	C13—H13	0.9800
O6—C21	1.439 (4)	C14—C20	1.523 (5)
O7—C1	1.373 (4)	C14—H14	0.9800
O7—C25	1.425 (4)	C15—C19	1.528 (5)
N1—C8	1.445 (5)	C15—H15	0.9800
N1—C11	1.466 (5)	C16—C17	1.490 (6)
N1—C7	1.480 (4)	C16—C18	1.501 (5)
N2—C12	1.498 (5)	C17—H17A	0.9600
N3—N4	1.312 (4)	C17—H17B	0.9600
N3—C22	1.360 (5)	C17—H17C	0.9600
N4—N5	1.351 (4)	C18—H18C	0.9600
N5—C23	1.326 (5)	C18—H18B	0.9600
N5—C24	1.451 (5)	C18—H18A	0.9600
C1—C2	1.382 (6)	C19—C20	1.521 (5)
C1—C6	1.390 (5)	C19—H19	0.9800
C2—C3	1.372 (6)	C20—H20	0.9800
C2—H2	0.9300	C21—C22	1.483 (6)
C3—C4	1.366 (6)	C21—H21A	0.9700
C3—H3	0.9300	C21—H21B	0.9700
C4—C5	1.377 (6)	C22—C23	1.366 (6)
C4—H4	0.9300	C23—H23	0.9300
C5—C6	1.385 (5)	C24—C25	1.506 (6)
C5—H5	0.9300	C24—H24A	0.9700
C6—C7	1.508 (5)	C24—H24B	0.9700
C7—C13	1.576 (5)	C25—H25A	0.9700
C7—H7	0.9800	C25—H25B	0.9700
			
C10—S9—C8	92.9 (2)	C12—C13—H13	107.8
C15—O3—C14	106.3 (3)	C7—C13—H13	107.8
C15—O4—C16	110.0 (3)	O3—C14—C20	101.8 (3)
C19—O5—C16	110.5 (3)	O3—C14—C13	110.0 (3)
C20—O6—C21	114.3 (3)	C20—C14—C13	117.5 (3)
C1—O7—C25	119.0 (3)	O3—C14—H14	109.0
C8—N1—C11	107.3 (3)	C20—C14—H14	109.0
C8—N1—C7	114.5 (3)	C13—C14—H14	109.0
C11—N1—C7	106.0 (3)	O4—C15—O3	111.0 (3)
O2—N2—O1	123.8 (4)	O4—C15—C19	105.4 (3)
O2—N2—C12	119.1 (4)	O3—C15—C19	106.9 (3)
O1—N2—C12	117.0 (4)	O4—C15—H15	111.1
N4—N3—C22	108.7 (4)	O3—C15—H15	111.1
N3—N4—N5	107.1 (3)	C19—C15—H15	111.1
C23—N5—N4	110.6 (4)	O5—C16—O4	104.6 (3)
C23—N5—C24	128.5 (4)	O5—C16—C17	108.9 (4)
N4—N5—C24	119.7 (4)	O4—C16—C17	109.0 (4)
O7—C1—C2	123.3 (4)	O5—C16—C18	111.7 (3)
O7—C1—C6	116.1 (3)	O4—C16—C18	109.5 (3)
C2—C1—C6	120.6 (4)	C17—C16—C18	112.7 (4)
C3—C2—C1	119.8 (4)	C16—C17—H17A	109.5
C3—C2—H2	120.1	C16—C17—H17B	109.5
C1—C2—H2	120.1	H17A—C17—H17B	109.5
C4—C3—C2	120.8 (4)	C16—C17—H17C	109.5
C4—C3—H3	119.6	H17A—C17—H17C	109.5
C2—C3—H3	119.6	H17B—C17—H17C	109.5
C3—C4—C5	119.2 (4)	C16—C18—H18C	109.5
C3—C4—H4	120.4	C16—C18—H18B	109.5
C5—C4—H4	120.4	H18C—C18—H18B	109.5
C4—C5—C6	121.7 (4)	C16—C18—H18A	109.5
C4—C5—H5	119.1	H18C—C18—H18A	109.5
C6—C5—H5	119.1	H18B—C18—H18A	109.5
C5—C6—C1	117.8 (4)	O5—C19—C20	110.9 (3)
C5—C6—C7	121.5 (3)	O5—C19—C15	104.4 (3)
C1—C6—C7	120.7 (3)	C20—C19—C15	104.1 (3)
N1—C7—C6	111.2 (3)	O5—C19—H19	112.3
N1—C7—C13	103.8 (3)	C20—C19—H19	112.3
C6—C7—C13	117.4 (3)	C15—C19—H19	112.3
N1—C7—H7	108.0	O6—C20—C19	109.3 (3)
C6—C7—H7	108.0	O6—C20—C14	110.1 (3)
C13—C7—H7	108.0	C19—C20—C14	101.6 (3)
N1—C8—S9	106.9 (3)	O6—C20—H20	111.8
N1—C8—H8B	110.4	C19—C20—H20	111.8
S9—C8—H8B	110.4	C14—C20—H20	111.8
N1—C8—H8A	110.4	O6—C21—C22	111.3 (3)
S9—C8—H8A	110.4	O6—C21—H21A	109.4
H8B—C8—H8A	108.6	C22—C21—H21A	109.4
C11—C10—S9	105.4 (3)	O6—C21—H21B	109.4
C11—C10—H10A	110.7	C22—C21—H21B	109.4
S9—C10—H10A	110.7	H21A—C21—H21B	108.0
C11—C10—H10B	110.7	N3—C22—C23	107.9 (4)
S9—C10—H10B	110.7	N3—C22—C21	121.3 (4)
H10A—C10—H10B	108.8	C23—C22—C21	130.3 (4)
N1—C11—C12	99.5 (3)	N5—C23—C22	105.7 (4)
N1—C11—C10	109.0 (3)	N5—C23—H23	127.2
C12—C11—C10	117.2 (4)	C22—C23—H23	127.2
N1—C11—H11	110.2	N5—C24—C25	109.8 (3)
C12—C11—H11	110.2	N5—C24—H24A	109.7
C10—C11—H11	110.2	C25—C24—H24A	109.7
N2—C12—C11	112.8 (3)	N5—C24—H24B	109.7
N2—C12—C13	113.8 (3)	C25—C24—H24B	109.7
C11—C12—C13	105.4 (3)	H24A—C24—H24B	108.2
N2—C12—H12	108.2	O7—C25—C24	106.5 (3)
C11—C12—H12	108.2	O7—C25—H25A	110.4
C13—C12—H12	108.2	C24—C25—H25A	110.4
C14—C13—C12	112.9 (3)	O7—C25—H25B	110.4
C14—C13—C7	117.0 (3)	C24—C25—H25B	110.4
C12—C13—C7	103.0 (3)	H25A—C25—H25B	108.6
C14—C13—H13	107.8		
			
C22—N3—N4—N5	0.6 (4)	N1—C7—C13—C12	8.4 (3)
N3—N4—N5—C23	−1.2 (4)	C6—C7—C13—C12	131.5 (3)
N3—N4—N5—C24	−169.7 (3)	C15—O3—C14—C20	43.5 (3)
C25—O7—C1—C2	15.5 (5)	C15—O3—C14—C13	168.8 (3)
C25—O7—C1—C6	−164.5 (3)	C12—C13—C14—O3	16.1 (4)
O7—C1—C2—C3	−179.6 (4)	C7—C13—C14—O3	135.5 (3)
C6—C1—C2—C3	0.4 (6)	C12—C13—C14—C20	131.9 (3)
C1—C2—C3—C4	−2.0 (6)	C7—C13—C14—C20	−108.7 (4)
C2—C3—C4—C5	0.9 (6)	C16—O4—C15—O3	−128.7 (3)
C3—C4—C5—C6	1.9 (6)	C16—O4—C15—C19	−13.3 (4)
C4—C5—C6—C1	−3.4 (6)	C14—O3—C15—O4	86.5 (3)
C4—C5—C6—C7	174.6 (4)	C14—O3—C15—C19	−28.0 (4)
O7—C1—C6—C5	−177.8 (3)	C19—O5—C16—O4	−22.5 (4)
C2—C1—C6—C5	2.2 (5)	C19—O5—C16—C17	−138.9 (4)
O7—C1—C6—C7	4.2 (5)	C19—O5—C16—C18	96.0 (4)
C2—C1—C6—C7	−175.8 (4)	C15—O4—C16—O5	22.0 (4)
C8—N1—C7—C6	79.3 (4)	C15—O4—C16—C17	138.3 (4)
C11—N1—C7—C6	−162.6 (3)	C15—O4—C16—C18	−97.9 (4)
C8—N1—C7—C13	−153.6 (3)	C16—O5—C19—C20	126.0 (3)
C11—N1—C7—C13	−35.5 (3)	C16—O5—C19—C15	14.4 (4)
C5—C6—C7—N1	47.2 (4)	O4—C15—C19—O5	−0.6 (4)
C1—C6—C7—N1	−134.8 (3)	O3—C15—C19—O5	117.6 (3)
C5—C6—C7—C13	−72.0 (5)	O4—C15—C19—C20	−116.9 (3)
C1—C6—C7—C13	105.9 (4)	O3—C15—C19—C20	1.2 (4)
C11—N1—C8—S9	−38.5 (3)	C21—O6—C20—C19	−110.0 (3)
C7—N1—C8—S9	78.8 (3)	C21—O6—C20—C14	139.3 (3)
C10—S9—C8—N1	16.8 (3)	O5—C19—C20—O6	156.1 (3)
C8—S9—C10—C11	8.6 (3)	C15—C19—C20—O6	−92.2 (3)
C8—N1—C11—C12	170.2 (3)	O5—C19—C20—C14	−87.7 (4)
C7—N1—C11—C12	47.5 (3)	C15—C19—C20—C14	24.1 (4)
C8—N1—C11—C10	47.0 (4)	O3—C14—C20—O6	75.0 (3)
C7—N1—C11—C10	−75.7 (4)	C13—C14—C20—O6	−45.2 (4)
S9—C10—C11—N1	−32.5 (4)	O3—C14—C20—C19	−40.6 (3)
S9—C10—C11—C12	−144.4 (3)	C13—C14—C20—C19	−160.8 (3)
O2—N2—C12—C11	83.3 (5)	C20—O6—C21—C22	−73.2 (4)
O1—N2—C12—C11	−92.5 (4)	N4—N3—C22—C23	0.1 (4)
O2—N2—C12—C13	−36.8 (5)	N4—N3—C22—C21	173.3 (3)
O1—N2—C12—C13	147.5 (4)	O6—C21—C22—N3	−69.4 (5)
N1—C11—C12—N2	−165.7 (3)	O6—C21—C22—C23	102.1 (5)
C10—C11—C12—N2	−48.5 (5)	N4—N5—C23—C22	1.2 (4)
N1—C11—C12—C13	−41.0 (3)	C24—N5—C23—C22	168.4 (4)
C10—C11—C12—C13	76.2 (4)	N3—C22—C23—N5	−0.8 (4)
N2—C12—C13—C14	−88.8 (4)	C21—C22—C23—N5	−173.1 (4)
C11—C12—C13—C14	147.1 (3)	C23—N5—C24—C25	−49.6 (5)
N2—C12—C13—C7	144.0 (3)	N4—N5—C24—C25	116.7 (4)
C11—C12—C13—C7	19.9 (4)	C1—O7—C25—C24	159.4 (3)
N1—C7—C13—C14	−116.0 (3)	N5—C24—C25—O7	−55.0 (4)
C6—C7—C13—C14	7.1 (5)		

### (II) (Z)-32,32-Dimethyl-27-nitro-25,26,27,27a,33a,35,36,36a-octahydro-21H,23H,61H-4,9-dioxa-2(5,6)-pyrrolo[1,2-c]thiazola-6(4,1)-triazola-3(5,6)-furo[2,3-d][1,3]dioxola-1(1,2)-benzenacyclononaphane Hydrogen-bond geometry (Å, º)

**Table:**

*D*—H···*A*	*D*—H	H···*A*	*D*···*A*	*D*—H···*A*
C23—H23···N3^i^	0.93	2.58	3.433 (6)	152
C25—H25*A*···N3^i^	0.97	2.60	3.553 (6)	168
C25—H25*B*···S9^ii^	0.97	2.80	3.591 (4)	140

Symmetry codes: (i) −*x*+1, *y*+1/2, −*z*+1; (ii) −*x*+2, *y*+1/2, −*z*+1.
